# Fibrin hydrogels are safe, degradable scaffolds for sub-retinal implantation

**DOI:** 10.1371/journal.pone.0227641

**Published:** 2020-01-13

**Authors:** Jarel K. Gandhi, Fukutaro Mano, Raymond Iezzi, Stephen A. LoBue, Brad H. Holman, Michael P. Fautsch, Timothy W. Olsen, Jose S. Pulido, Alan D. Marmorstein

**Affiliations:** Department of Ophthalmology, Mayo Clinic, Rochester, Minnesota, United States of America; University of Florida, UNITED STATES

## Abstract

Retinal pigment epithelium (RPE) transplantation for the treatment of macular degeneration has been studied for over 30 years. Human clinical trials have demonstrated that RPE monolayers exhibit improved cellular engraftment and survival compared to single cell suspensions. The use of a scaffold facilitates implantation of a flat, wrinkle-free, precisely placed monolayer. Scaffolds currently being investigated in human clinical trials are non-degradable which results in the introduction of a chronic foreign body. To improve RPE transplant technology, a degradable scaffold would be desirable. Using human fibrin, we have generated scaffolds that support the growth of an RPE monolayer *in vitro*. To determine whether these scaffolds are degraded *in vivo*, we developed a surgical approach that delivers a fibrin hydrogel implant to the sub-retinal space of the pig eye and determined whether and how fast they degraded. Using standard ophthalmic imaging techniques, the fibrin scaffolds were completely degraded by postoperative week 8 in 5 of 6 animals. Postmortem histologic analysis confirmed the absence of the scaffold from the subretinal space at 8 weeks, and demonstrated the reattachment of the neurosensory retina and a normal RPE–photoreceptor interface. When mechanical debridement of a region of native RPE was performed during implantation surgery degradation was accelerated and scaffolds were undetectable by 4 weeks. These data represent the first *in situ* demonstration of a fully biodegradable scaffold for use in the implantation of RPE and other cell types for treatment of macular degeneration and other retinal degenerative diseases.

## Introduction

Age-related macular degeneration (AMD) is the leading cause of blindness in the developed world, with an estimated 196 million cases globally by 2020 [1]. AMD causes a loss of central, high acuity vision, and is thought to result from dysfunction of the RPE. The concept of treating AMD and other forms of macular degeneration using RPE transplantation has been studied since the late 1980s [2,3]. Yet, until recently, few clinical trials had been conducted due to lack of an abundant RPE cell source. Pluripotent stem cells offer an abundant cell source with multiple studies demonstrating reliable differentiation into RPE cells [4–7]. A number of recent clinical trials to treat AMD or inherited macular degeneration using stem cell derived RPE cells support the potential efficacy of this approach [8–11].

Since RPE cells are no longer a rate limiting factor, the field has more recently shifted focus to develop a practical means of delivering RPE cells into the sub-retinal space. The first clinical trial of embryonic stem cell-derived RPE (ES-RPE) used the simple approach of injecting a single bolus cell suspension into the sub-retinal space [8]. Though safe, this study had a low transplant integration and viability [8,12]. The adaptation of tissue engineering principles led to 3 recent engraftment successes in clinical trials for AMD. The first study to demonstrate the use of induced pluripotent stem cell (iPSC)-derived cells utilized a gelatin scaffold to generate an RPE monolayer that is enzymatically released from the scaffold prior to delivery as a free-floating monolayer [9,13]. The one patient who received an autologous transplant showed stabilization of her visual acuity [9], with the transplant surviving as long as 4 years [14]. The RPE in the autologous transplant exhibited folding and wrinkling resulting in an excessively thick RPE layer in the absence of a scaffold that may have contributed to some of the long term complications associated with this transplant [14]. The other studies utilized synthetic scaffolds, polyethylene terephthalate (PET) [10] and Parylene [11], to support an ES-RPE monolayer throughout the transplantation surgery, and implanted the scaffold together with the RPE. In these two studies the scaffold remained intact after 1 year of followup. The combined 6 patients receiving non-degradable scaffolds showed similar visual stability, with one who received the PET-based transplant reporting improvement in visual function [10,11]. These early studies have mainly demonstrated proof-of-principal for transplants on non-degradable scaffolds. While the RPE in these studies reportedly survive out to 1 year, the persistence of the non-degradable scaffold may become problematic. In animal studies where non-degradable scaffolds are implanted in the subretinal space there is significant damage to the overlying retina in the absence of RPE cells [10,15]. As such, while RPE transplant shows great promise, there are is a substantial opportunity to improve these procedures by developing a degradable scaffold that will not introduce a chronic foreign body, yet permit precision placement of a flat RPE monolayer.

In discussing potential degradable scaffolds, it is important to recognize that a number of groups are also studying the transplantation of stem cell-derived photoreceptor cells (PR) for retinal degenerative diseases such as retinitis pigmentosa. In a similar evolutionary course, the initial models of delivering suspended PR have been markedly improved with the use of scaffolds [16]. The use of synthetic scaffolds containing cylindrical holes for the progenitor differentiation to PR has been proposed to generate PR arrays as a therapeutic [17]. Though the two concepts utilize different cell populations for different mechanisms of blindness, both approaches can benefit from a similar implant delivery approach. For example, whether delivering an RPE monolayer or photoreceptor sheet, both implants must be placed in the sub-retinal space, which lies between the neurosensory retina and the choroid-Bruch’s membrane-RPE tissue complex (CBR). Many popular materials with the potential for degradation have been reported as potential scaffolds for use in retinal cell transplantation [17–22]. However, to date, none of these materials has been demonstrated to degrade completely when placed in the subretinal space of the eye. Because of the unique micro-environment of the sub-retinal space [23], it is not reasonable to accept that the degradation and safety profiles will be similar for these materials in the subretinal space compared to that in other parts of the eye or body.

Previously, we had investigated fibrin hydrogels for use as scaffolds in RPE transplantation [24]. Fibrin provides the matrix for formation of a clot during the natural wound healing process. As such, the body possesses enzymes both to form and breakdown fibrin clots [25]. Fibrin is approved by the FDA as a glue for use in surgery and products currently on the market (e.g. Evicel and Tisseel) have demonstrated records of safety in the eye and brain after several decades of clinical use [26–28]. Previously, we generated fibrin gels with appropriate geometry, suitable mechanical properties, and degradation kinetics for use in RPE transplantation [24]. These fibrin scaffolds support the growth and differentiation of iPSC-RPE monolayers that exhibit the characteristics associated with an RPE phenotype [24], which was maintained *in vitro* following scaffold degradation. Herein, we investigated the degradation of cell-free fibrin hydrogel implants within the subretinal space. We observed that degradation of the scaffolds occurs within 8 weeks in the subretinal space and that the implant causes no damage to the surrounding tissues. Furthermore, we observed that the time course for degradation can be accelerated to <4weeks by mechanical debridement of the native RPE.

## Materials and methods

### Gel fabrication

**Fig 1:**
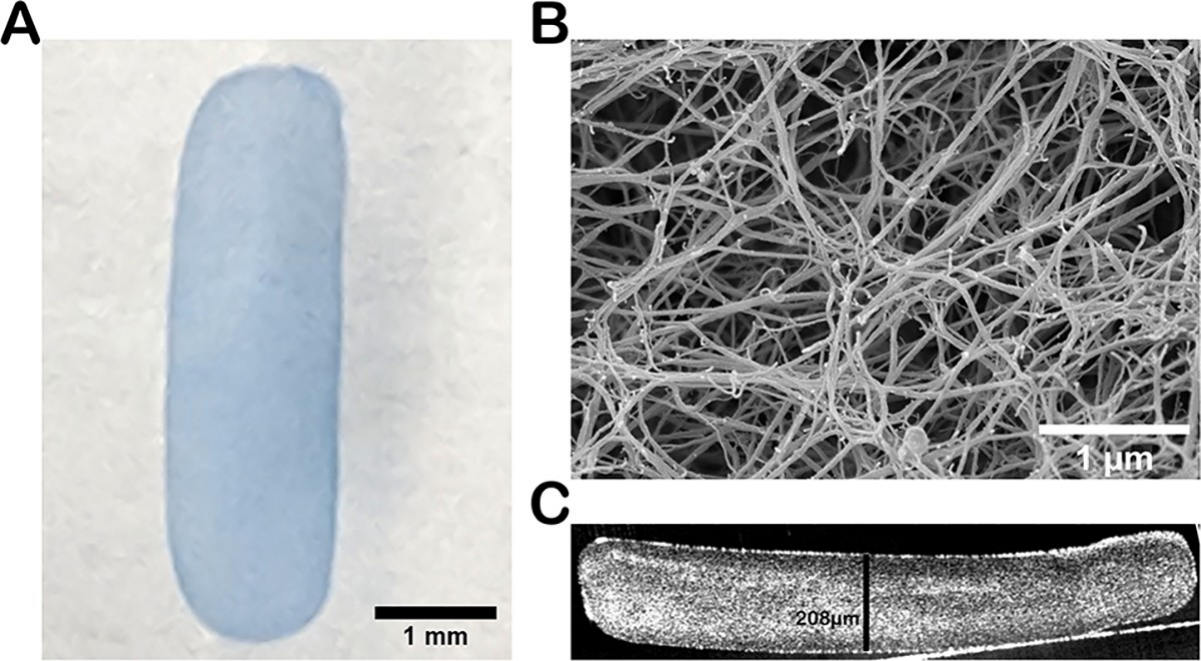
Fibrin hydrogel implant characterization. (A) Brightfield image of a 1.5mm x 5.0mm fibrin implant colored with trypan blue (B) Scanning electron micrograph of the top surface of the fibrin implant showing characteristic crosslinking fibrils. (C) Optical coherence tomography (OCT) of the fibrin implant in cross-section, demonstrates uniform fibril formation and thickness.

Fibrin hydrogels were formed using the Evicel (Ethicon; Somerville, NJ) fibrin glue kit as previously described [24] at a final mixing concentration of 40 mg/mL Fibrinogen and 33 U/mL thrombin. The mixture was placed in a custom mold to achieve a thickness of 200μm and incubated for 2 hrs at 37°C to achieve gelation. The gel was then hydrated in PBS for a minimum of 1 hr at 37°C. Individual implants were obtained by using a custom punch of 1.5mm wide x 5.0mm long, to achieve the desired geometry of an oblong shaped implant with rounded edges (Fig 1A). Implants were stained in 0.04% trypan blue solution (Gibco; Waltham, MA) to facilitate visualization during the surgical and post-operative examination. Following production, implants were stored in phosphate buffered saline (PBS) at room temperature.

### Characterization of fibrin hydrogels

Scanning electron microscopy (SEM) was performed as previously described [24]. Briefly, gels were fixed overnight in 2.5% paraformaldehyde and 1% glutaraldehyde in 0.1M phosphate buffer pH 7 containing 1.0mM MgCl_2_ and 0.13 mM CaCl_2_. Gels were then processed and imaged at the Mayo Microscopy and Cell Analysis Core using a Hitachi S-4700 (Hitachi High Technologies; Schaumburg, IL) Scanning Electron Microscope.

Optical coherence tomography was used to evaluate the thickness and consistency of the implants. OCT was performed as before [24] using an Envisu R220 (Leica; Wetzlar, Germany) set up using an AIM table, with the camera and attached telecentric lens faced down toward the gel which was in PBS. B scans were taken of the gels and analyzed using the accompanying InVivoVue software (Leica).

### Implantation device prototype

**Fig 2:**
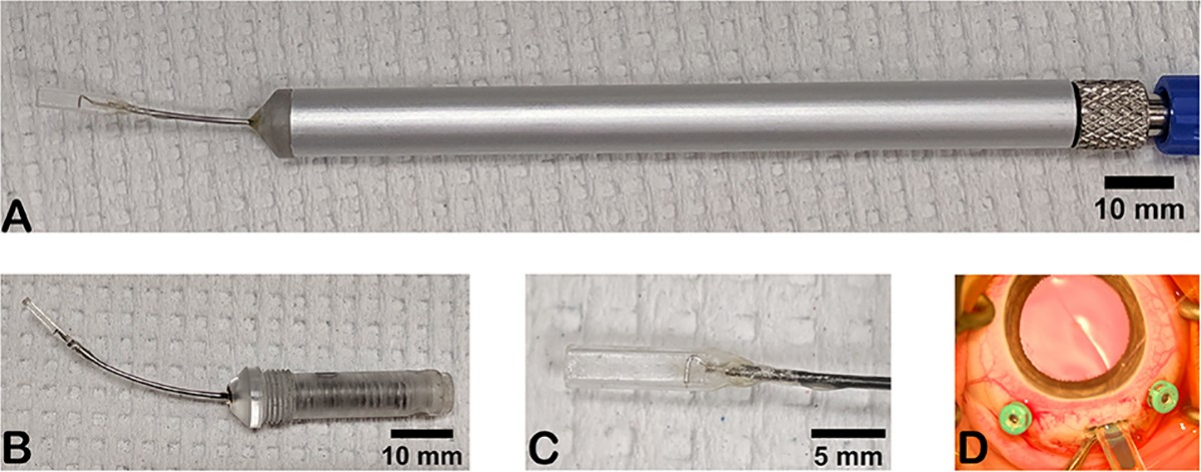
Sub-retinal implantation device prototype. (A) Photo of the device. The disposable tip assembly (B) is screwed into the metal (silver) handle. The actuator resides within the handle and is not visible. The blue luer lok tubing connector is attached to the actuator connector. (B) Image of the disposable tip assembly without the handle. (C) Close up image of the clear plastic housing. The wire plunger is visible within the clear plastic housing. (D) Surgical video screenshot showing the device entering through the sclera. The blue implant is visible within the device.

An implantation device was prototyped with assistance from Elite Custom Solutions (Rochester, MN). The device consists of a reusable handle and a disposable tip assembly (Fig 2). The reusable handle has a pneumatic actuator, luer-lock connector, and threads for the disposable tip assembly. The disposable tip assembly consists of a spring-guided pin attached to a metal wire plunger that is housed through a stainless steel tube hub and within a clear plastic housing tip. The clear plastic housing tip was generated using a 3D printing stereolithography technique and the material used was WaterShed XC 11122. The fibrin hydrogel implant is loaded within the clear plastic housing of the tip prior to implantation. To deploy the implant from the housing, pressure is applied via syringe to the pneumatic actuator, activating the wire plunger.

### Animals

We used female domestic (Yorkshire) pigs (Manthei Hog Farm; Elk River, MN), with starting weights of 22-35kg. Animals were pre-screened for exclusion criteria, including cupped optic disk, iris neovascularization, and pre-existing retinal detachment or cataract. All experiments were approved by the Mayo Clinic IACUC and adhered to the ARVO statement for the use of animals in ophthalmic and vision research.

### Surgery

Animals were initially anesthetized with 5mg/kg Telazol and 2mg/kg Xylazine intramuscular followed by 0.18mg/kg Buprenorphine SR intramuscular prior to surgery. Once fully anesthetized, the pupil was dilated with 2.5% phenylephrine and 1% tropicamide eye drops and 0.5% proparacaine drops were administered for topical analgesia. Following intubation, sedation was maintained with inhalant Isoflurane (1–5%) throughout the surgery.

Following a temporal canthotomy and placement of a pediatric ophthalmic speculum, the conjunctiva was incised, and a standard 25ga, 4-port vitrectomy (Accurus System: Alcon; Ft Worth, TX) was performed. Following a subtotal vitrectomy with a posterior vitreous detachment (PVD), a serous retinal detachment of 6-10mm diameter was created using a 38ga (MedOne; Sarasota, Fl) or 40ga cannula (Incyto; South Korea). A small area of retina was cauterized with a 25ga diathermy probe (Alcon) at the inferior border of the retinal detachment and a retinotomy was created using 25ga vertical vitreoretinal scissors (Beaver-Visitec; Waltham, MA). In some cases, mechanical debridement of the endogenous RPE under the detachment was performed using a beveled soft tip aspiration cannula (MedOne). A 3.6mm scleral incision was then made approximately 3.5 mm posterior to the corneal limbus using a 20ga MVR knife (Alcon). The choroid was cauterized and incised using a 3.4mm satin knife (Alcon). The custom implantation device, loaded with the fibrin implant, was then inserted through the scleral incision and aligned with the retinotomy and the implant ejected into the subretinal space. The implantation device was then withdrawn, and the sclerotomy sutured. In some cases, perfluorocarbon liquid (Alcon) was used to flatten the retina over the implant. Fluid/air exchange and tamponade with silicone oil (Alcon) or SF_6_ gas (Alcon) was applied at the discretion of the surgeon. Following removal of the ports, the animal was administered a single dose of 300mg of Cefazolin in the sub-Tenon space. Topical 0.3% Gentamycin twice daily for 4 days and subcutaneous 4mg/kg Carprofen once daily for 4 days was also administered.

### Post-operative exams

During postoperative exams, pigs were anesthetized with 0.1mg/kg dexmedetomidine and 5mg/kg Ketamine. Pupils were dilated with 2.5% phenylephrine and 1% tropicamide eye drops. Proparacaine drops (0.5%) were administered for topical analgesia. If the animal had poor eye tracking for imaging, the animal was also intubated, and sedation maintained with inhalant Isoflurane (1–5%). After imaging was complete, an anesthetic reversing agent, 1mg/kg Atipamezole, was given intramuscularly.

Indirect ophthalmoscopy was used to examine the fundus. Next, color fundus (Topcon TRC-NW200; Tokyo, Japan), OCT images (OptoVue iVue 1.0; Fremont, CA), and fluorescein angiography (FA) dosed at 1mg/Kg using a handheld fundus camera (Volk Optical; Mentor, OH) were used to image the implantation site. Imaging was performed weekly or bi weekly in all animals until euthanasia. OCT image analysis was performed on multiple sections through the center of the implant zone.

Animals were sacrificed at 2, 4, 8 and 12 weeks (Table 1) using intravenous pentobarbital (390mg/mL). One animal was also sacrificed at each of the following time points: time zero (immediately post-operative), 1 day and 2 days after implantation. Following sacrifice, eyes were enucleated and fixed in 10% neutral buffered formalin for a minimum of 72hrs and processed through paraffin. Ten μm sections were cut and stained using hematoxylin and eosin as described [29]. Eyes from 0, 1 and 2 day time points, were embedded in frozen tissue embedding medium (Tissue-Tek; Torrance, CA). 10μm cryo-sections were used *in lieu* of paraffin to preserve as much of the retinal architecture as possible.

**Table 1:** Table of study animals.

Case #	Sacrifice timepoint	Fully degraded?	Notes
1	2 day	No	
2	1 day	No	
3	0 day	No	
4	10 week	Yes	
5	8 week	Yes	
6	8 week	Yes (by 1 week)	
7	8 week	Yes	
8	2 week	No	
9	2 week	No	
10	4 week	No	
11	12 week	Yes (by 8 weeks)	
12	2 week	Yes	
13	5 week	Yes (by 4 weeks)	Debridement
14	8 week	Yes	
15	4 week	Yes	Intentional Debridement
16	2 week	No	Intentional Debridement

### Statistics

Data was analyzed using JMP 14 (SAS; Cary, NC). For OCT measure of retinal detachment thickness over time, a two-way ANOVA was used. After ANOVA analysis, significance was tested amongst groups using a Tukey HSD and/or student t-test.

## Results

### Fibrin gels

Fibrin gels (Fig 1A) were manufactured as previously described to a thickness of 200 μm [24]. Our prior work showed that this thickness is ideal to generate a gel stiff enough to maneuver with various surgical tools without bending or folding or impeding the path of movement [24]. Gels had the characteristic fibrin nano fibril crosslinked structure on SEM imaging (Fig 1B). Imaging with OCT verified that implants used in this study had an average thickness of 202.5 ± 18.9 μm with an average surface area of 6.2 ± 0.2 mm^2^, an average width of 1.45 ± 0.02 mm, and average length of 4.71 ± 0.03 mm (n = 6).

### Implantation device prototype

We developed a novel insertion device to implant fibrin scaffolds (Fig 2A & 2B). The device has a rectangular housing tip (Fig 2C) geometrically optimized to house the implant. The outer dimensions of the tip were minimized to facilitate a small (3.6mm) sclerotomy. To minimize hand-placement instability, we used a pneumatic-driven actuator that did not require finger manipulation. This allowed compatibility with the viscous fluid injection module of common surgical vitrectomy systems, which can be triggered by a foot pedal. A rectangular, transparent housing tip allowed visualization of the implant (Fig 2D). Visibility was considered important to permit the surgeon to verify proper polarity of the implant and deployment without damage to the implant.

The housing tip had outer dimensions of 2.3mm width and 1.0mm height, with inner dimensions of 1.9mm width and 0.6mm height (S1 Fig). The stainless steel hub was curved to parallel the inner surface of the pig eye with a radius of 45mm (S1 Fig).

### Implantation surgery

**Fig 3:**
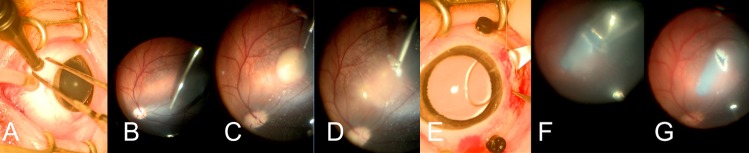
Montage of surgical procedure. (A) Placement of the valved entry ports. (B) Vitrectomy. Triamcinolone is used as a contrast agent to perform the posterior vitreous detachment. (C) A subretinal bleb is created using a 40ga cannula. (D) A 1.8mm retinotomy is created using 25ga vertical scissors. The retina was pre-cauterized using a diathermy probe to prevent bleeding. (E) A 3.6mm wide sclerotomy is created using a slit knife. (F) The implantation device is used to place the fibrin implant into the subretinal space. The blue implant is visible under the retina.

We settled on a surgical model compatible with current vitreoretinal surgical practice. This includes a trans-vitreal, trans-retinal approach to gain access to the subretinal space (Fig 3, S1 Video). This was accomplished by creating a retinal detachment and retinotomy following vitrectomy. To minimize the size and damage created by the retinotomy, we utilized a small implant (1.5mm width) that was sufficient to span the diameter of the human fovea [30] and proved favorable due to the highly vascular retina found in the pig eye. The pig retina has an area centralis that contains fewer large vessels and contains a higher density of cone photoreceptors [31]. Successful placement of the fibrin implant into the area centralis was achieved in 16 pigs.

### Post-operative live imaging follow-up

Sixteen surgeries were performed. Three of these included mechanical debridement of the RPE. Twelve surgeries had fluid/air exchange, one received a silicone oil tamponade and two received ~20% SF_6_ gas tamponade. In cases with only fluid/air exchange, the air resorbed by day 2–3 post-operative. In cases with 20% SF_6_ gas tamponade, the gas resorbed completely by post-operative day five. In cases with partial gas in the globe, imaging or a full indirect exam were not possible. In the case with the silicone oil tamponade, both OCT and fundus images were difficult to obtain, and indirect exam was the preferred method of examination. Implant degradation rates did not differ between eyes with gas or silicone tamponade.

**Fig 4:**
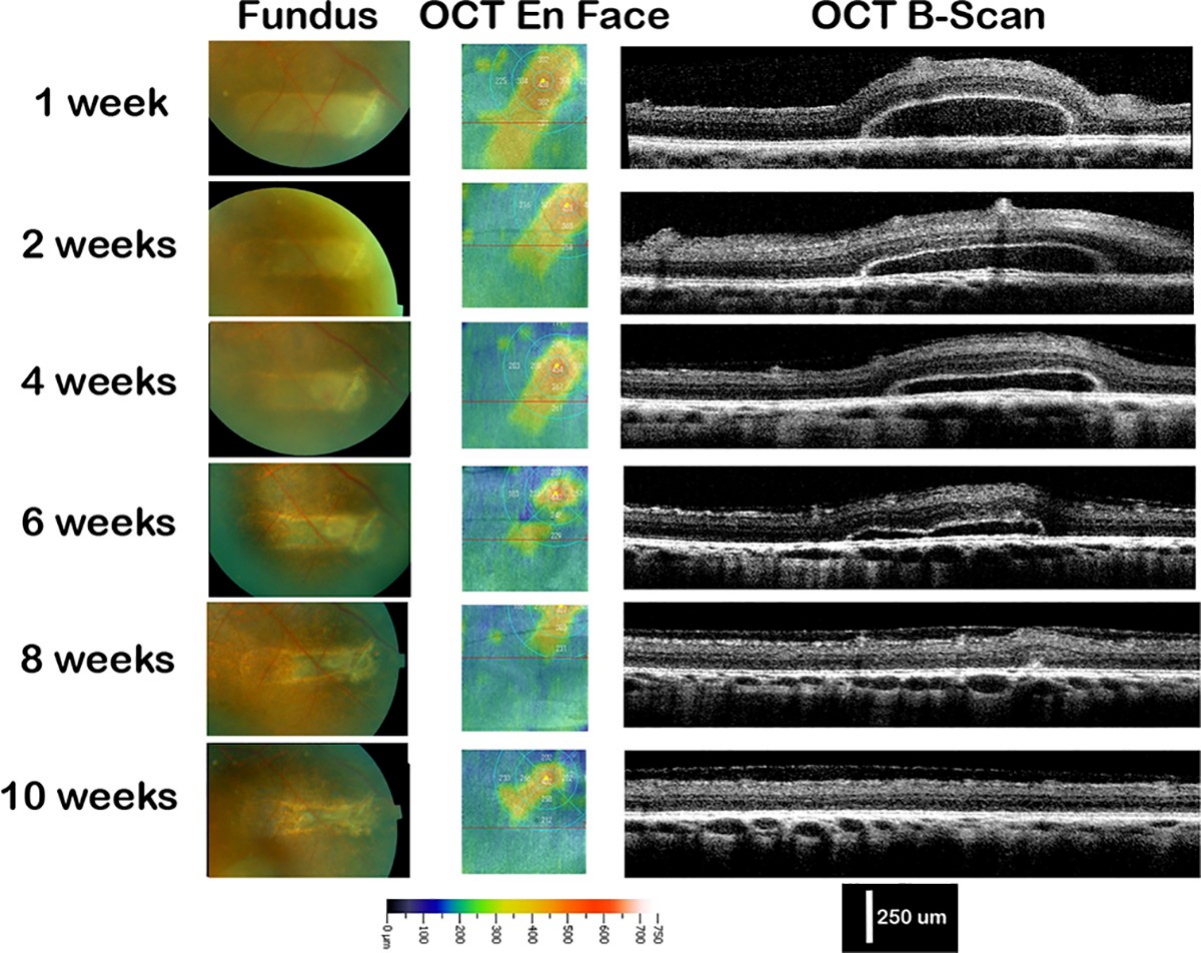
Live post-operative imaging. Time course of fundus and OCT en face and b-scan images for animal #11 from 1 week to 10 weeks post-operative showing serial degradation. On the OCT en face images, the circle is centered to the retinotomy site for comparable areas between images. The red line indicates the region of the b-scan. The color intensity scale represents the rough thickness of the retina on the en face image and is the same for all images. The scale bar applies to all OCT b-scan images.

**Fig 5:**
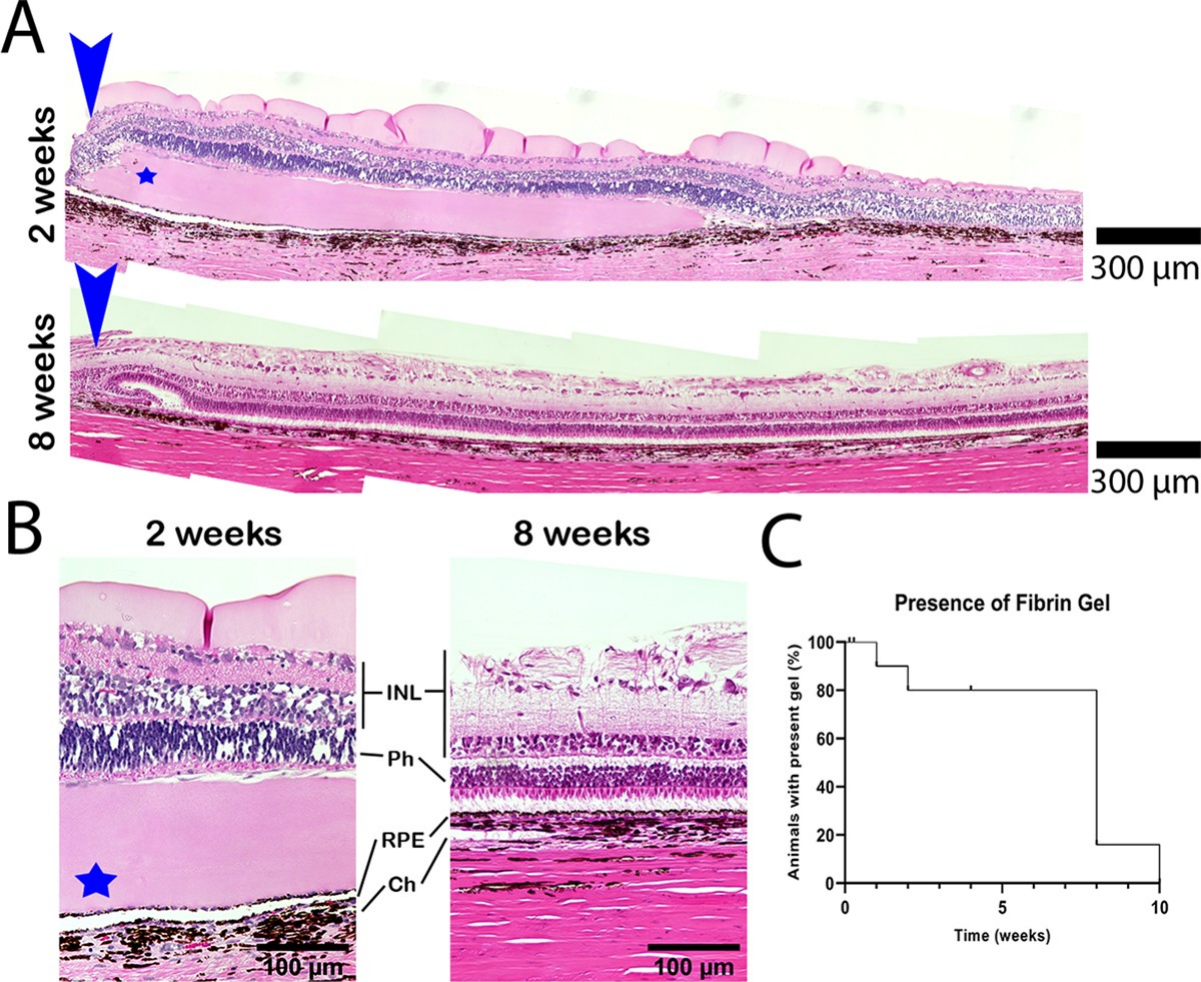
Fibrin implantation histology. (A) Photomicrograph of H&E stained histological sections from animals #12 (2 weeks) and #7 (8 weeks). The blue arrow indicates the retinotomy. The fibrin implant appears eosinophilic (blue star), with the bulk of the gel remaining at 2 weeks. By 8 weeks, there is no evidence of the fibrin gel. The neural retina within the implanted region appears healthy at both time points. The retinotomy appears to thicken over time. (B) Close up images of regions where the implant was placed showing the healthy retina. The blue star indicates the fibrin gel. In the 8 week timepoint, the inner retina appears thicker because of the use of silicone oil as a tamponade. (C) Kaplan-meier graph showing the percent of animals within the cohort with evidence of remaining fibrin. In most cases, the fibrin implant is completely degraded by 8 weeks. INL: Inner nuclear layer. Ph: Photoreceptor layer. RPE: Retinal pigment epithelium. Ch: Choroid.

Fibrin scaffolds were visualized with indirect ophthalmoscopy and OCT at week one (Fig 4). Using OCT, a rectangular gap between the retina and underlying choroid-Bruchs membrane-RPE (CBR) tissue was observed (Fig 4). This was later confirmed by histology as the fibrin implant (Fig 5A). In contrast to our *in vitro* work where the implant was hyper-reflective under OCT, the implant was hypo-reflective using OCT *in vivo*. This is likely due to the relatively higher reflective properties of the retinal tissue in comparison to fibrin and the necessity to adjust contrast accordingly.

Of the 13 animals receiving fibrin scaffold implants in which RPE were not debrided, all showed signs of fibrin degradation over time (Fig 4). The OCT images showed a raised plateau that completely resolved by week eight (Fig 4) without damage to the retina or CBR. The retinotomy closed in all cases and led to a raised scar.

### Histology

H&E stained and unstained cryosections were examined at 0, 1, & 2 days post-implantation to confirm the presence of the scaffold in the subretinal space (S2 Fig). At later time points, H&E stained paraffin sections of enucleated eyes were examined to confirm the degradation of the fibrin gel and general health of the retina in the region of the implant (Fig 5A & 5B). The retinotomy and optic nerve head were used as points of reference to locate the region of the implant. A scar was noted at the retinotomy that was thicker than the surrounding retina (Fig 5A, blue arrow). At two weeks post-implantation, an eosinophilic hydrogel is visible within the sub-retinal space, distal to the retinotomy site. The neurosensory retina over the gel appears to be in good health with normal anatomy and photoreceptor outer segments in contact with the fibrin hydrogel (Fig 5A & 5B). There was no evidence of rosette formation or immune cell infiltration (n = 2, 2 weeks). The outer surface of the fibrin appears to remain mostly smooth. Signs of fibrin degradation were observed. These were mainly confined at 2 weeks to a tapering of the outer edges of the gel.

Table 1 shows a summary of the animals included in the study. Because 3 animals were kept alive longer than the complete degradation of the implant, a Kaplan-Meier survival analysis was used to demonstrate presence of fibrin degradation (Fig 5C). At 1 week, 91.7% of the cohort had remaining evidence of the fibrin implant. At 2, 4, and 8 weeks the percent cohort with presence of the fibrin implant were 80.2%, 80.2%, 16.0% respectively. By 10 weeks, no fibrin implant could be identified (Fig 5C, Table 1).

### RPE debridement accelerates fibrin degradation

In an animal with accidental RPE damage, indirect ophthalmoscopy and OCT indicated the absence of the fibrin gel by week four. Histology at week five, confirmed both the absence of the fibrin scaffold and RPE atrophy in the region receiving the implant. Histology also showed significant thickening of the retina above the RPE atrophy region and an extended localized retinal detachment as might be expected in the absence of RPE cells. This led us to hypothesize that the debridement of endogenous RPE could result in acceleration of fibrin gel degradation.

**Fig 6:**
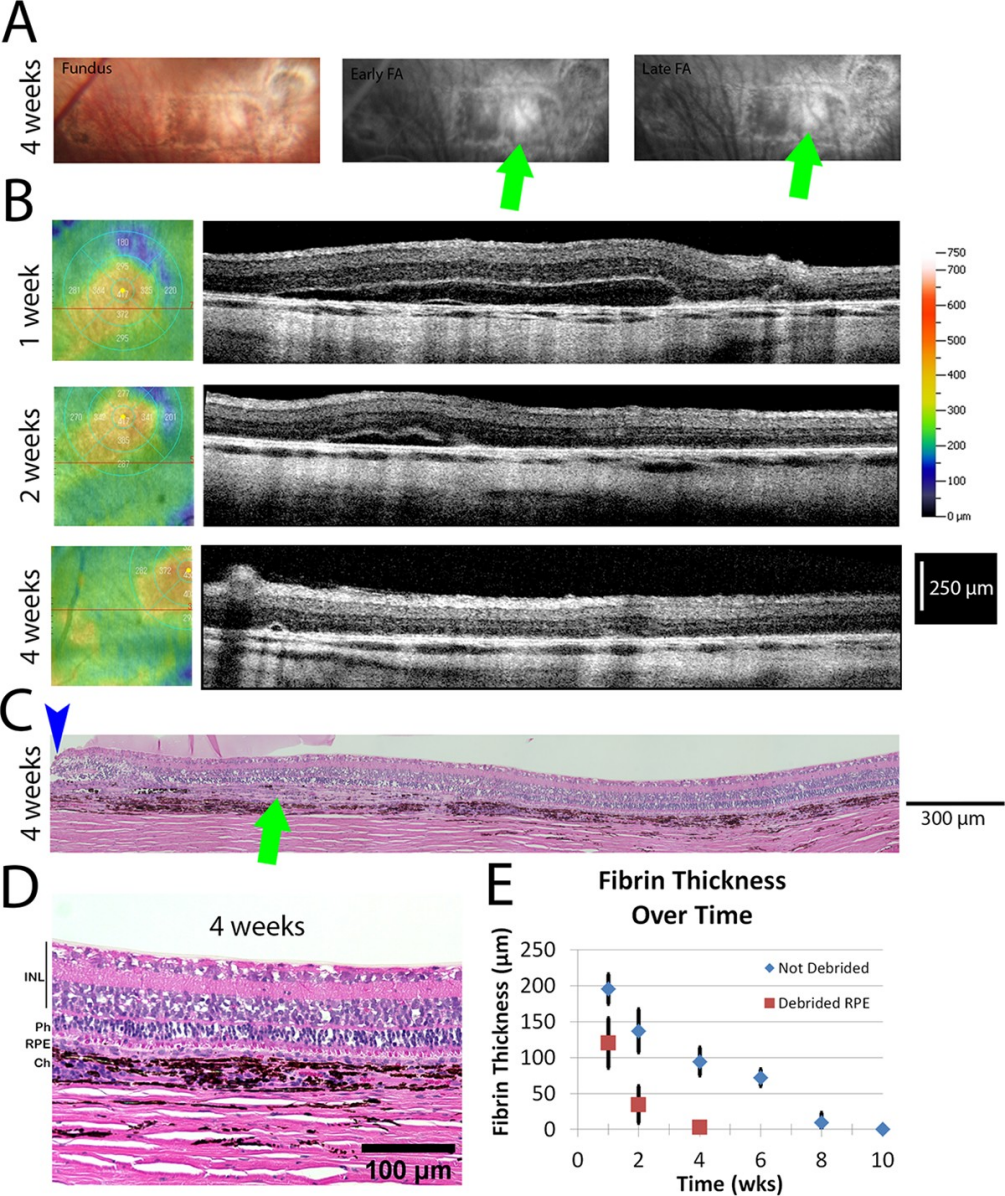
Faster degradation due to RPE debridement. (A) Fundus and fluorescein angiogram (FA) images (early and late timepoints) showing the site of implantation. A window defect is seen in both early and late FA images, indicating the region of RPE debridement. (B) OCT en face and b-scan images for animal #15 from 1 week to 4 weeks post-operative. (C) Image of H&E stained histological sections from animal #13. The blue arrow indicates the retinotomy site and the green arrow indicates the RPE debrided region. (D) Close up image of region where the implant was placed showing the healthy retina. (E) Graph showing the thickness of the retinal detachment in OCT images over time for animals with and without debridement. The thickness is suggestive of the remaining fibrin implant. There is a statistically significant difference between the debrided and not debrided groups (p<0.001). INL: Inner nuclear layer. Ph: Photoreceptor layer. RPE: Retinal pigment epithelium. Ch: Choroid.

To test the hypothesis that removal of the native RPE may accelerate fibrin degradation, we mechanically debrided a region of the endogenous RPE using a beveled soft tip aspiration cannula in 2 pigs. Debridement was verified postoperatively using fluorescein angiography and OCT. The site of debridement appeared hyper-fluorescent in fluorescein angiograms, while the areas with endogenous RPE were hypo-fluorescent (Fig 6A). The hyper-fluorescent area demonstrated RPE disruption without any fibrotic changes or choroidal neovascularization when examined using OCT (Fig 6A & 6B).

Over time, the fibrin implant degraded at a faster pace as observed via OCT and fundus exam (Fig 6B). Histology at 4 weeks confirmed no evidence of the implant (Fig 6C). To quantify the degradation, measurements of fibrin gel thickness were made from multiple OCT images of a representative animal of each condition through the center of the implant region as a function of time (Fig 6E). Without debridement, the thicknesses of the gel at 1, 2, 4, 6, 8, and 10 weeks were 195.2±20.6μm, 136.8±29.9um, 94.4±19.2 μm, 71.8±11.8 μm, 9.8±13.4 μm, and 0 μm, respectively (mean ± sd, pig #11, p<0.001) (Fig 6E). With debridement, the gel thicknesses at 1, 2, and 4 weeks were 120±34.7 μm, 34.4±25.7 μm, and 2.8±6.3 μm, respectively (mean ± sd, pig #15, p<0.001) (Fig 6E). ANOVA analysis confirms a statistically significant difference between the two animals with and without debridement (p<0.001).

## Discussion

In this study, we found that a surgically implanted fibrin scaffold placed in the sub-retinal space consistently degraded within 8 weeks, without damage to the neurosensory retina or endogenous RPE. After the scaffold was degraded, the retina appears to reattach to the underlying RPE. When the RPE was mechanically debrided, the implant was degraded within 4 weeks. To the best of our knowledge, this is the first report that demonstrates complete degradation of a scaffold within the sub-retinal space.

The surgical device was optimized for use in the pig eye. Future iterations of the device will attempt to minimize the outer dimensions of the rectangular housing to minimize the scleral incision by decreasing the inner width and wall thickness. For prospective human use, this device would also require adjusting the curvature to better match the geometry of the human eye.

Fibrin has a long history of safe use for a variety of applications in the clinic [25,32]. This includes ocular use for corneal and conjunctival leaks, sclerotomy closure, and retinal breaks [27,28,32]. Our implant uses the same formulation as the fibrin glue currently used in the clinic. Both poly lactic-glycolic acid (PLGA) and polycaprolactone (PCL) also have long histories of safe use in similar applications [33,34]. For example, dissolvable sutures made of PLGA and PCL are routinely used in the clinic and in the eye for sclerotomy closure [35]. However, it is very important to note that the subretinal space is a unique environment [23]. It is immune privileged and has a microenvironment that differs from the general interstitium. Because of this unique microenvironment, it is not appropriate to assume that the degradation of a given material will mimic that observed for other applications in other areas of the body. As such, there is a general lack of data verifying the degradation rate of any scaffold placed in the subretinal space, or the variables that might affect that rate of degradation. For example, recent animal studies assessing sub-retinal transplantation of scaffolds formed from PLGA or PCL failed to establish that the scaffold was completely degraded [18,20], possibly due to the extended time required for complete degradation.

Our previous work has shown that the RPE are capable of degrading the fibrin scaffold in vitro over the course of days [24]. This is prevented using anti-fibrinolytic compounds such as aprotinin. While the overall aim of this transplant effort is to place cells within the sub-retinal space, the scope of the present study was narrowed to understand the degradation and safety of the fibrin hydrogel alone. We hypothesize that the introduction of RPE and absence of an anti-fibrinolytic would accelerate the rate of fibrin scaffold degradation in the subretinal space. The introduction of the RPE with the implant however, creates a xenogeneic transplant that will trigger an immune response. That response could have confounded our efforts to determine the feasibility and time course of fibrin scaffold degradation in the subretinal space. Future studies will address the effects of RPE cells and immunosuppression of fibrin scaffold degradation.

Slow, long-term degradation has the same potential concerns for foreign body response and long-term inflammation that non-degradable scaffolds present. Though degradable, a lengthy time course of degradation may elicit an inflammatory response sufficient to affect the transplanted RPE. For example, others have shown the development of an epiretinal membrane through contact of PLGA with the inner retinal layer [36]. In a recent study that examined the use of PLGA to transplant iPSC-RPE monolayers in pigs, it is difficult to assess any *in vivo* immunologic response to the scaffold material because of the use of aggressive immunosuppression used [18]. A second concern is that the degradation byproducts of the scaffold may alter the local environment or accumulate to toxic levels over time. Both PLGA and PCL are known to have acidic byproducts, which in the case of PLGA, has previously been shown to cause photoreceptor death [19]. The group using PCL for photoreceptor transplant has cited this as their reason to switch from PLGA to PCL [20], though PCL too generates acidic byproducts. Thus, we argue that stating a material is degradable is not sufficient, but defining a time course and its influence on safety within the targeted environment is important.

For the fibrin scaffolds we tested, the complete degradation and anatomic restoration of the RPE/photoreceptor interface argues against long term toxicity, even in animals where degradation required a full 8 weeks. These data are only applicable to scaffolds of the same volume, density, and thickness used in the current study. While outside the scope of the current study, we would predict that changes to those parameters would alter degradation accordingly. Still, it would be interesting to identify the mechanisms that could accelerate or slow the rate of degradation as humans and swine may differ in this regard. For example, humans have a 20-fold higher plasma concentration of the fibrinolytic enzyme precursor plasminogen, compared to swine [37]. As such, fibrin scaffold degradation may occur substantially faster in humans. What we observed, and tested, was that debridement of the native RPE accelerated fibrin degradation, cutting the time to within 30 days. We hypothesize that this occurs by allowing for greater access of plasminogen in the interstitial fluid to the implant region due to the absence of RPE in the debrided region. In a small number of animals without intentional debridement of endogenous RPE, we observed rapid degradation of the scaffold (Fig 5C, Table 1). It is possible that we caused small areas of debridement that were not observed in those eyes. Interestingly, later stages of AMD are characterized by patchy RPE loss, suggesting that the environment may be more similar to RPE debrided animals and would favor accelerated fibrin degradation in humans compared to pigs. Future clinical studies are required to understand the rate of degradation of fibrin scaffolds in humans, though such a scaffold would be studied in combination with RPE cells. *In vitro*, iPSC derived RPE cells will rapidly degrade the scaffold unless anti-fibrinolytic agents such as aprotinin are added to the cell culture medium [24]. How the presence of cells on the scaffold affects degradation *in vivo* remains to be determined.

In conclusion, our data demonstrate that fibrin is a degradable, safe material for sub-retinal transplantation. Fibrin implants degraded within 8 weeks of implantation with no signs of damage to the neural retina or underlying CBR. Debridement of the native RPE accelerated the degradation to 4 weeks post implantation. These data support the use of fibrin scaffolds for the transplantation of RPE and potentially PRs which also would require placement in the subretinal space.

## Supporting information

S1 Data Raw files of quantitative data. Raw excel files of quantitative data used in the manuscript.
(ZIP)

S1 Fig Technical drawings of implantation device. (A) Photomicrograph of rectangular housing tip with outer dimensions of 2.3mm width and 1.0mm height and inner dimensions of 1.9mm width and 0.6mm height. (B) Photomicrograph of disposable tip loaded into handle. The radius of curvature of the stainless steel hub was 45mm.
(TIF)

S2 Fig 24hr fibrin implantation histology. Photomicrograph of H&E stained cryo sections from animal #2 (1 day). The fibrin implant appears eosinophilic (blue star), with proper placement within the sub-retinal space. Following fixation, the eye was processed through 30% (w/v) sucrose in PBS, which resulted in dehydration of the fibrin hydrogel causing it to appear thinner.
(TIF)

S1 Video Surgical procedure of sub-retinal implantation. Video of surgical procedure.
(MP4)
