# Supplementary figures and images for: Fibrin hydrogels are safe, degradable scaffolds for sub-retinal implantation
Source: PLoS One. 2020 Jan 13;15(1):e0227641. doi: 10.1371/journal.pone.0227641 (PMC6957177; doi:10.1371/journal.pone.0227641)

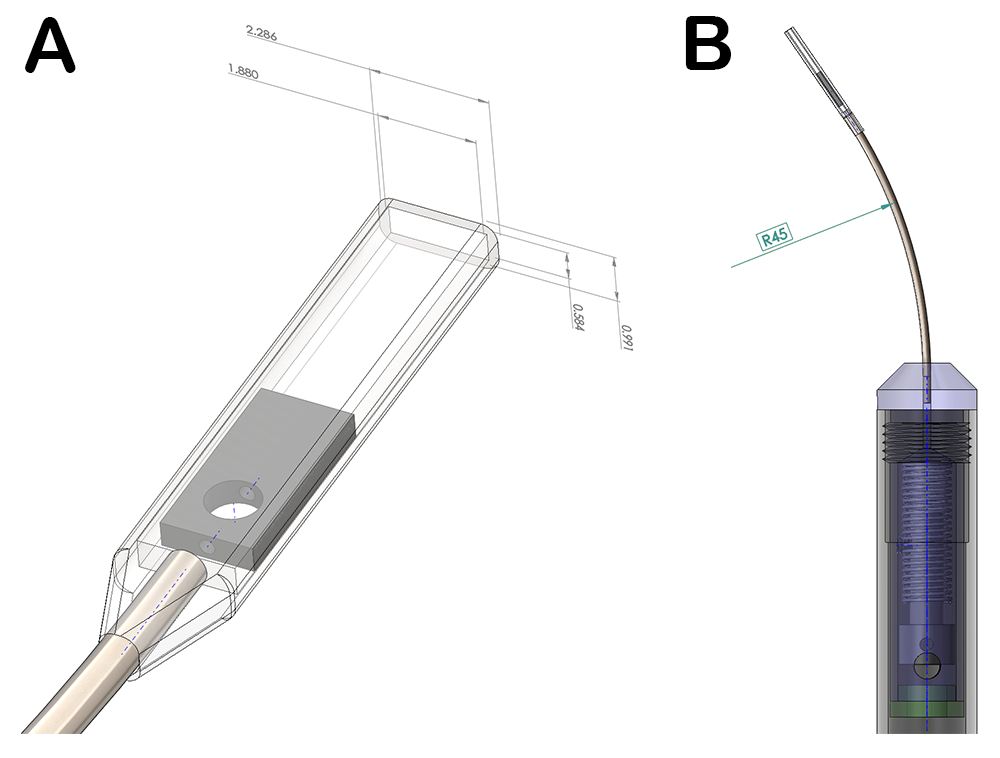

Supplement: S1 Fig — (A) Photomicrograph of rectangular housing tip with outer dimensions of 2.3mm width and 1.0mm height and inner dimensions of 1.9mm width and 0.6mm height. (B) Photomicrograph of disposable tip loaded into handle. The radius of curvature of the stainless steel hub was 45mm. (TIF) [file pone.0227641.s002.tif]

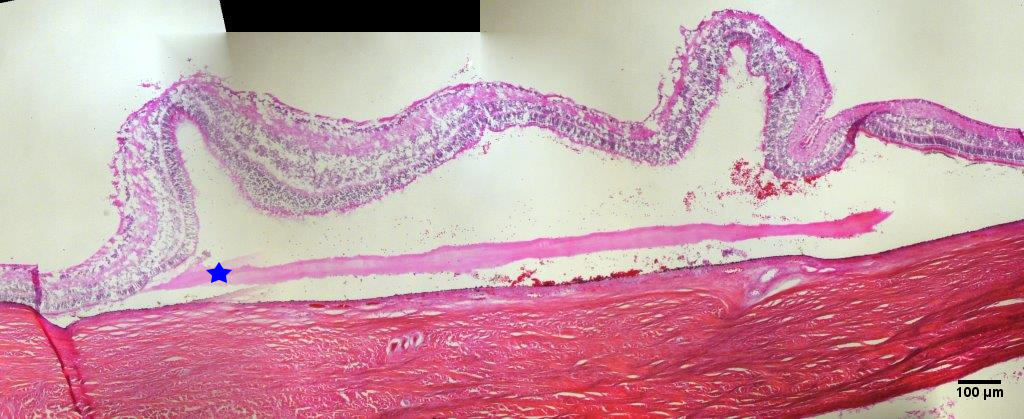

Supplement: S2 Fig — Photomicrograph of H&E stained cryo sections from animal #2 (1 day). The fibrin implant appears eosinophilic (blue star), with proper placement within the sub-retinal space. Following fixation, the eye was processed through 30% (w/v) sucrose in PBS, which resulted in dehydration of the fibrin hydrogel causing it to appear thinner. (TIF) [file pone.0227641.s003.tif]
